# Shedding of OXA-181 carbapenemase-producing *Escherichia coli* from companion animals after hospitalisation in Switzerland: an outbreak in 2018

**DOI:** 10.2807/1560-7917.ES.2019.24.39.1900071

**Published:** 2019-09-26

**Authors:** Aurélien Nigg, Michael Brilhante, Valentina Dazio, Mathieu Clément, Alexandra Collaud, Stefanie Gobeli Brawand, Barbara Willi, Andrea Endimiani, Simone Schuller, Vincent Perreten

**Affiliations:** 1 Institute of Veterinary Bacteriology, Bern, University of Bern; 2 Graduate School of Cellular and Biomedical Sciences, Bern, University of Bern; 3 Department of Clinical Veterinary Medicine, Bern, University of Bern; 4 Clinic for Small Animal Internal Medicine, University of Zurich, Zurich, Switzerland; 5 Institute for Infectious Diseases, University of Bern, Bern, Switzerland

**Keywords:** dogs, cats, antibiotic resistance, carbapenem, veterinary hospital

## Abstract

**Background:**

Carbapenem-resistant Enterobacteriaceae pose a serious threat to public health worldwide, and the role of companion animals as a reservoir is still unclear.

**Aims:**

This 4-month prospective observational study evaluated carriage of carbapenem-resistant Enterobacteriaceae at admission and after hospitalisation in a large referral hospital for companion animals in Switzerland.

**Methods:**

Rectal swabs of dogs and cats expected to be hospitalised for at least 48 h were taken from May to August 2018 and analysed for the presence of carbapenem-resistant Enterobacteriaceae using selective agar plates. Resistant isolates were further characterised analysing whole genome sequences for resistance gene and plasmid identification, and ad hoc core genome multilocus sequence typing.

**Results:**

This study revealed nosocomial acquisition of *Escherichia coli* harbouring the carbapenemase gene *bla*_OXA-181_, the pAmpC cephalosporinase gene *bla*_CMY-42_ as well as quinolone resistance associated with *qnrS1* and mutations in the topoisomerases II (GyrA) and IV (ParC). The *bla*_OXA-181_ and *qnrS1* genes were identified on a 51 kb IncX3 plasmid and *bla*_CMY-42_ on a 47 kb IncI1 plasmid. All isolates belonged to sequence type ST410 and were genetically highly related. This *E. coli* clone was detected in 17 of 100 dogs and four of 34 cats after hospitalisation (21.6%), only one of the tested animals having tested positive at admission (0.75%). Two positive animals were still carriers 4 months after hospital discharge, but were negative after 6 months.

**Conclusions:**

Companion animals may acquire carbapenemase-producing *E. coli* during hospitalisation, posing the risk of further dissemination to the animal and human population and to the environment.

## Introduction

Companion animals nowadays not only share the owner's home environment but also benefit from intensive veterinary care in case of serious illness. Veterinary referral hospital environments are similar to human hospitals in that animal patients face similar risks of developing nosocomial infections.

Bacterial infections in dogs and cats are frequently treated with critically important antimicrobials such as fluoroquinolones or cephalosporins. In very rare cases, even the last resort antibiotics of human medicine such as carbapenems may be used in companion animals in Switzerland to treat infections refractory to any other standard antimicrobial used in veterinary medicine (Ordinance on Veterinary Medicinal Products, SR 812.212.27, Art. 6).

Such treatments pose the risk of selecting resistance to these classes of antibiotics in Enterobacteriaceae through the acquisition of plasmid-mediated cephalosporinase genes (e.g. *bla*_CTX-M_, *bla*_CMY_) and fluoroquinolones resistance genes (e.g. *qnr*, *aac-(6)-Ib-cr*), but also through chromosomal mutations in the promotor region of the AmpC β-lactamase and in the quinolone resistance-determining region (QRDR) of the topoisomerases II (GyrA) and IV (ParC) [1,2]. Resistances to carbapenems have also emerged in *Escherichia coli* and *Klebsiella pneumoniae* [3]. Three main groups of carbapenemases are referenced according to the Ambler classification: class A (e.g. KPC), class B (IMP, VIM, NDM) and class D (e.g. OXA) [4]. Among the OXA carbapenemases, OXA-48-like ones have been described as phantom carbapenemases since they may be difficult to detect using phenotypic susceptibility testing [5].

Until now, animals were considered to play a minor role in the dissemination of OXA-48-like carbapenemases [6], but reports of sporadic infections in dogs caused by carbapenemase-producing (CP) Enterobacteriaceae in Europe [7,8], in the United States (US) [9] and in Northern Africa [10] suggest that companion animals may represent an unsuspected reservoir of carbapenem-resistant bacteria. Recently, hospitalised companion animals in Italy were found to acquire carbapenem-resistant bacteria such as *Acinetobacter* spp. [11]. Nonetheless, very little is known about carriage and dynamics of CPE in dogs and cats before and after hospitalisation.

Here, we characterised by whole genome sequencing (WGS) CP *E. coli* isolated from cats and dogs at admission and after hospitalisation to a large veterinary referral hospital in Switzerland, identifying their antimicrobial resistance genes (ARG) and mobile genetic elements (MGE) as well as the phylogenetic relationship among the isolates.

## Methods

### Study design and sampling

This study was part of a larger prospective study which included three large veterinary referral clinics and two smaller veterinary practices in Switzerland. The present prospective observational study was performed at a university referral hospital with a 24/7 emergency service. The hospital provides advanced medical care for 6,000 dogs and cats annually. Dogs and cats were included irrespective of their underlying problems and previous treatments if they were expected to be hospitalised for at least 48 h and the owner gave informed consent. All animals presented here were recruited via the emergency service of the hospital. During the enrolment period of 4 months (1 May–31 August 2018), ca 2,000 animals were treated at the hospital and 134 of them fulfilled the study criteria and were screened.

Rectal swabs were collected in awake animals at admission to the hospital and at discharge, using polyurethane foam culture swabs with Ames transport medium (BBL CultureSwabs). If available, animals positive at discharge were retested once or twice within 36 to 166 days after their discharge of the clinic.

### Isolation and identification of the strains

Swabs were placed into 5 mL of Luria-Bertani (LB) broth for overnight enrichment at 37 °C. A loopful of the culture was streaked onto CHROMID OXA-48 and CHROMID CARBA (Biomérieux SA, Marcy-l'Étoile, France) selective plates, which were incubated at 37 °C for 24 h under aerobic conditions.

Colonies were sub-cultivated onto trypton soy agar plates containing 5% sheep blood (TSA-S) (Becton and Dickinson Company, Franklin Lakes, US) and identified to the species level by matrix-assisted laser desorption/ionisation time-of-flight mass spectrometry (MALDI-TOF MS) (Bruker Daltonics GmbH, Bremen, Germany).

### Biochemical and molecular tests

Carbapenemase production was detected using the Blue-Carba test [12]. Before WGS, carbapenemase-producing isolates were tested for the presence of *bla*_OXA-48-like_ genes by PCR [5].

### Antimicrobial susceptibility testing

Minimal inhibitory concentrations (MIC) of 16 antimicrobials (ampicillin, cefepime, cefotaxime, ceftazidime, chloramphenicol, ciprofloxacin, colistin, ertapenem, gentamicin, imipenem, meropenem, nalidixic acid, sulfamethoxazole, tetracycline, tigecycline and trimethoprim) were determined by broth microdilution using Sensititre EUVSEC and EUVSEC2 plates (Thermo Fisher Scientific, Waltham, US) and following the guidelines from the European Committee on Antimicrobial Susceptibility Testing (EUCAST) [13]. MIC results were interpreted using the EUCAST criteria, except for nalidixic acid, sulfamethoxazole and tetracycline, for which criteria from the Clinical and Laboratory Standards Institute (CLSI) were used [14].

### Whole genome sequencing and in silico analysis

Total DNA from OXA-48-like-positive strains was isolated using the DNeasy Blood and Tissue Kit (Qiagen, Venlo, the Netherlands), and purified using the AMPure XP paramagnetic bead-based chemistry (Beckmann Coulter, Brea, US). All CP *E. coli* were sequenced (2 × 150 bp paired-end) using the Illumina HiSeq technology (Illumina Inc, San Diego, US) at the Eurofins Institut (Konstanz, Germany). The genomic DNA of strain AR24.2b was additionally sequenced with MinION on a R9.4 Spot On flow cell using the library preparation kit SQK-LSK108 (Oxford Nanopore Technologies (ONT), Oxford, United Kingdom (UK)). Genome assembly of all strains was performed using SPAdes (v3.12.0) and the obtained contigs were used for in silico analysis. The genome assembly of strain AR24.2b was performed by Unicycler (v0.4.4), using both long (ONT) and short reads (Illumina), as well as by CANU (v1.7) that only uses the ONT long reads. The obtained scaffolds were polished by read-mapping with paired-end Illumina reads using Pilon (v1.22) and with Geneious software v10.1.3 (Biomatters Ltd, Auckland, New Zealand). Annotation was performed with PROKKA v1.12. The paired-end reads of all CP *E. coli* strains were mapped to the final annotated plasmid sequences of AR24.2b. Online tools available at the Center for Genomic Epidemiology (Technical University of Denmark DTU, Lyngby, Denmark) were used to detect known ARG (ResFinder 3.0) to determine incompatibility (Inc) groups of the plasmids, (PlasmidFinder 1.3) and for multilocus sequence typing (MLST 2.0). Transposable elements present in the plasmids were identified using ISfinder (https://isfinder.biotoul.fr/). Blast ring image generator (BRIG) was used to generate circular map comparisons of plasmids (based on BLASTn) [15]. Comparison with closely related plasmids from the National Center for Biotechnology Information (NCBI) was achieved using the NCBI tool BLASTn (https://blast.ncbi.nlm.nih.gov/Blast.cgi, accessed November 2018).

### Phylogenetic analyses

An ad hoc core genome multilocus sequence typing (cgMLST) scheme was created using SeqSphere+ (v. 401, Rindom GmbH, Münster, Germany). The *E. coli* of sequence type (ST) 410 from a dog in the UK (GenBank accession number CP031653) was defined as the reference genome from which 4,177 coding regions of genes were extracted by the cgMLST Target Finder v1.4. The contigs obtained from SPAdes of all 24 CP *E. coli* in this study, together with the reference genome and with six other genomes of *E. coli* ST410 isolated from humans or from the environment and published in GenBank (GenBank accession numbers CP029630, CP027205, CP026473, CP024801, CP023899 and CP018965), were loaded and searched for the 4,177 gene targets using built-in BLAST. Overall, 3,778 genes were shared among all isolates, which we defined as the core genome. The visualisation of the phylogenetic distance was achieved using the allelic profile of all strains to generate a neighbour-joining tree using SeqSphere+ with the parameters ‘pairwise ignoring missing values; % columns difference’ for the distance calculation.

### Ethical statement

The study protocol was approved by the national ethics committee (BE 16/18) and signed informed consent was obtained from the owners before enrolment of the animals in the study.

## Results

### Occurrence of carbapenemase-producing *Escherichia coli*

Rectal swabs were taken from 100 dogs and 34 cats at admission to a large veterinary referral clinic in Switzerland between 1 May and 31 August 2018. Carriage of CP *E. coli* was detected in only one dog and none of the cats at admission (0.75%; 95% confidence interval (CI): 0–2.2).

In contrast, 17 of 76 dogs and four of 21 cats that were resampled at discharge were found to harbour CP *E. coli* (21.6%; 95% C: 13.4–29.8) indicating nosocomial acquisition of the bacteria. The 37 remaining animals were not available for resampling (n = 22) or deceased (n = 15).

**Figure 1:**
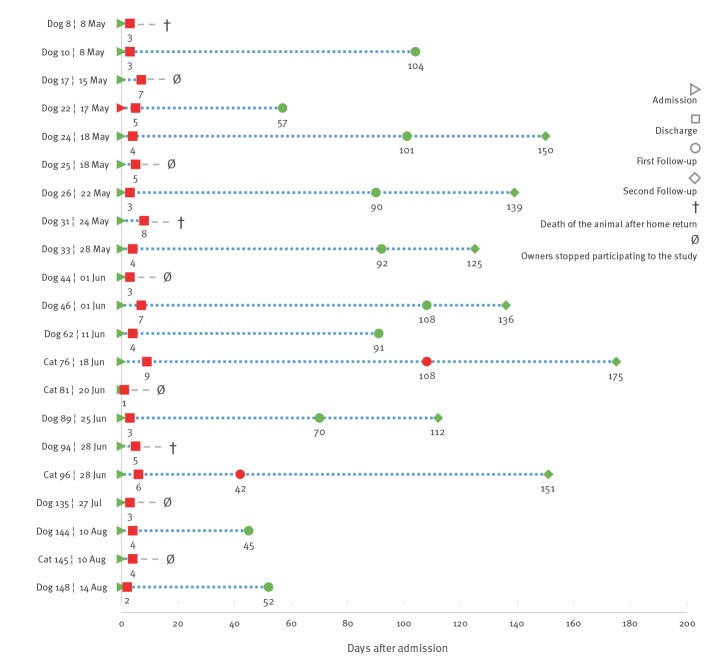
Temporal acquisition and carriage of carbapenemase-producing *Escherichia coli* ST410, Switzerland, May–August 2018 (n = 24)

The dog positive for CP *E. coli* at admission was not under antimicrobial treatment at arrival and had no previous history of hospitalisation at this clinic, but whether it was previously hospitalised in another clinic is not known. Nor was it the first animal tested positive during the study period. This dog was still positive at discharge (Figure 1).

Results as detected at admission and discharge to a companion animal clinic, as well as once or twice within 175 days after hospitalisation.

Shapes in green: test negative for CP *E. coli* carriage; shapes in red: test positive for CP *E. coli* carriage. Blue dotted line: time line of screened animals; grey dashed line: animals which were not resampled.

The demographics, hospitalisation details and antimicrobial treatments of the CP *E. coli*-carrying animals are summarised in Table 1. Animals were presented to the emergency service of the hospital with a wide range of complaints. All cats and 13 of the 17 dogs spent time in the intensive care unit of the hospital. The majority of dogs (14/17) and cats (3/4) were treated with antimicrobials during their hospitalisation; however, none of them received carbapenems.

**Table 1:** Demographic, hospitalisation and antimicrobial treatment details of dogs and cats positive for OXA181-producing *Escherichia coli* ST410, Switzerland, May–August 2018 (n = 21)

Parameter	Dogs (n = 17)	Cats (n = 4)
Age median in years (IQR)	7.0 (4.0–9.0)	4.5 (2.3–10.5)
Weight median in kg (IQR)	16.6 (7.8–34)	4.2 (3.0–5.7)
Sex		
Female (entire/neutered)	9 (5/4)	2 (0/2)
Male (entire/neutered)	8 (6/2)	2 (1/1)
Diagnoses		
Gastrointestinal disease	7	1
Neurological disease	5	0
Urinary tract disease	2	2
Other	3	1
Hospitalisation days; median (IQR)	4 (3–5)	5 (1.8–8.3)
ICU days; median (IQR)	3 (1–5)	2.3 (1.5–8.6)
Antimicrobial pre-treatment^a^		
Yes	4	1
No	11	3
Unknown	2	0
Antimicrobial treatment during hospitalisation		
Yes	14	3
No	3	1
Antimicrobials used during hospitalisation^b^		
Ampicillin/sulbactam	9	3
Cefazolin	1	1
Clindamycin	2	0
Doxycycline	1	0
Enrofloxacin	1	0
Metronidazole	1	0
Sulfamethoxazole/trimethoprim	1	0
Amoxicillin/clavulanic acid	1	0

ICU: intensive care unit; IQR: interquartile range.

^a^ Antimicrobials used for pre-treatments (i.e. prior to presentation/referral) were amoxicillin/clavulanic acid (n = 2), amoxicillin (n = 1), enrofloxacin (n = 1) and metronidazole (n = 1).

^b^ Includes mono- and combination therapy.

Follow-up samples were obtained within 36 to 101 days after discharge from 12 of 21 CP *E. coli*-positive animals. The other nine animals could not be retested because they had died (n = 3), or because the owners withdrew their participation in the study (n = 6). Two cats still carried CP *E. coli* 36 days and 99 days after discharge (Figure 1). Seven of the 12 animals that underwent a first follow-up could be retested for a second time within a time interval of 109 to 166 days after discharge of the clinic, including the two cats that were still positive after the first follow-up. None of them were found to be shedding CP *E. coli* at the second follow-up (Figure 1).

### Phylogenetic analyses

**Figure 2:**
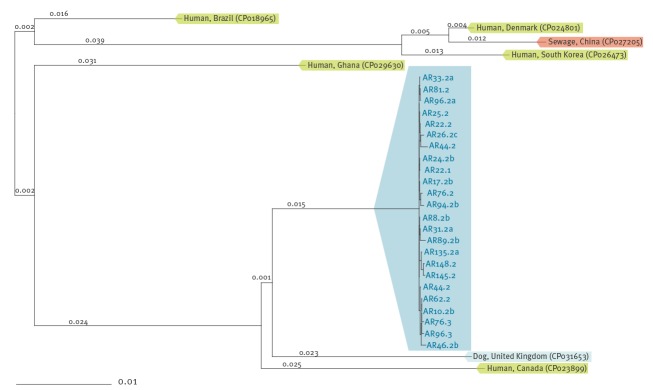
Phylogenetic neighbour-joining tree of all carbapenemase-producing *Escherichia coli* of ST410 isolated from animals hospitalised in a companion animal clinic (n = 24) and others available in GenBank (n = 7), Switzerland, May–August 2018

All 24 CP *E. coli* detected in this study belonged to ST410. Analysis of the 24 strains using cgMLST confirmed that they were all highly related, all clustering in the same branch of the phylogenetic tree, in contrast to the sequences of seven other and independent *E. coli* ST410 from animal, environmental and human origin. The most closely related strain was a canine *E. coli* isolated in the UK in 2018, which carried the carbapenemase gene *bla*_NDM-5_, the plasmid-mediated AmpC (pAmpC) cephalosporinase gene *bla*_CMY-42_, and the β-lactamase gene *bla*_TEM-190_ [16] (GenBank accession number CP031653) (Figure 2).

The tree was generated and drawn using SeqSphere+ (v. 401, Rindom GmbH, Münster, Germany) for the comparison of 3,778 genes from the core genome (cgMLST) of the genomic sequences.

Blue: isolates from animals; green: isolates from humans; orange: isolates from the environment.

*E. coli* isolates used for the comparison were carrying the following resistance genes to β-lactams, cephalosporins and carbapenems: CP018965: *bla*_KPC-2_, *bla*_OXA-1_, *bla*_CTX-M-15_; CP023899: *bla*_CTX-M-15_, *bla*_TEM-1B_; CP024801: *bla*_CMY-2_, *bla*_NDM-5_, *bla*_TEM-1B_, *bla*_CTX-M-15_, *bla*_OXA-1_; CP027205: *bla*_CMY-2_, *bla*_TEM-1B_, *bla*_CTX-M-65_, *bla*_NDM-5_; CP026473: *bla*_CMY-2_, *bla*_NDM-5_, *bla*_TEM-1B_, *bla*_CTX-M-15_, *bla*_OXA-1_, *bla*_OXA-181_; CP029630: *bla*_TEM-1A_, *bla*_OXA-9_, *bla*_CTX-M-15_, *bla*_NDM-1_; CP031653: *bla*_NDM-5_, *bla*_CMY-42_, *bla*_TEM-190_.

### Antimicrobial resistance profile of the carbapenemase-producing *E. coli*

All CP *E. coli* exhibited the same antimicrobial resistance profile; they were non-susceptible to ampicillin, cefepime, cefotaxime, ceftazidime, ciprofloxacin, ertapenem and nalidixic acid, except for one isolate (AR144.2) which was susceptible to ceftazidime and resistant to tetracycline (Table 2).

**Table 2:** Minimum inhibitory concentrations of 16 antibiotics for carbapenemase-producing *Escherichia coli* isolates of ST410 from cats and dogs, Switzerland, May–August 2018 (n = 24)

MIC of antibiotics in μg/mL^a^ and resistance breakpoints^b^ in parentheses			AMP (> 8)	CHL (> 8)	CIP (> 0.5)	COL (> 2)	ETP (> 0.5)	FEP (> 4)	FOT (> 2)	GEN (> 4)	IMI (> 4)	MERO (> 8)	NAL (≥ 32)	SMX (≥ 512)	TAZ (> 4)	TET (≥ 16)	TGC (> 2)	TMP (> 4)
Strain	Animal	Time point of isolation																
**AR8.2b**	Dog 8	Discharge	**> 64**	≤ 8	**> 8**	≤ 1	**> 2**	**8**	**> 4**	≤ 0.5	1	0.5	**> 128**	16	**> 128**	4	0.5	0.5
**AR10.2b**	Dog 10	Discharge	**> 64**	≤ 8	**> 8**	≤ 1	**> 2**	**8**	**> 4**	≤ 0.5	0.5	0.5	**> 128**	32	**> 128**	4	≤ 0.25	1
**AR17.2b**	Dog 17	Discharge	**> 64**	≤ 8	**> 8**	≤ 1	**> 2**	**8**	**> 4**	≤ 0.5	0.5	0.5	**> 128**	16	**> 128**	4	0.5	0.5
**AR22.1**	Dog 22	Admission	**> 64**	≤ 8	**> 8**	≤ 1	**> 2**	**8**	**> 4**	≤ 0.5	1	0.5	**> 128**	16	**> 128**	4	0.5	0.5
**AR22.2**	Dog 22	Discharge	**> 64**	≤ 8	**> 8**	≤ 1	**> 2**	**16**	**> 4**	1	0.5	0.5	**> 128**	16	**> 128**	4	0.5	0.5
**AR24.2b**	Dog 24	Discharge	**> 64**	≤ 8	**> 8**	≤ 1	**2**	**8**	**> 4**	≤ 0.5	0.5	0.25	**> 128**	32	**> 128**	4	≤ 0.25	2
**AR25.2**	Dog 25	Discharge	**> 64**	≤ 8	**> 8**	≤ 1	**2**	**8**	**> 4**	≤ 0.5	0.5	0.5	**> 128**	16	**> 128**	8	0.5	1
**AR26.2c**	Dog 26	Discharge	**> 64**	≤ 8	**> 8**	≤ 1	**> 2**	**8**	**> 4**	≤ 0.5	1	0.5	**> 128**	16	**> 128**	4	0.5	1
**AR31.2a**	Dog 31	Discharge	**> 64**	≤ 8	**> 8**	≤ 1	**> 2**	**8**	**> 4**	≤ 0.5	0.5	0.5	**> 128**	16	**> 128**	4	0.5	1
**AR33.2a**	Dog 33	Discharge	**> 64**	≤ 8	**> 8**	≤ 1	**2**	**8**	**> 4**	≤ 0.5	0.5	0.5	**> 128**	16	**> 128**	4	0.5	1
**AR44.2**	Dog 44	Discharge	**> 64**	≤ 8	**> 8**	≤ 1	**2**	**8**	**> 4**	≤ 0.5	0.5	0.5	**> 128**	16	**> 128**	4	0.5	0.5
**AR46.2b**	Dog 46	Discharge	**> 64**	≤ 8	**> 8**	≤ 1	**2**	**8**	**> 4**	≤ 0.5	0.5	0.25	**> 128**	16	**> 128**	4	≤ 0.25	1
**AR62.2**	Dog 62	Discharge	**> 64**	≤ 8	**> 8**	≤ 1	**2**	**8**	**> 4**	≤ 0.5	0.5	0.5	**> 128**	16	**> 128**	4	0.5	1
**AR76.2**	Cat 76	Discharge	**> 64**	≤ 8	**> 8**	≤ 1	**2**	**8**	**> 4**	≤ 0.5	0.5	0.5	**> 128**	16	**> 128**	4	0.5	1
**AR81.2**	Cat 81	Discharge	**> 64**	≤ 8	**> 8**	≤ 1	**> 2**	**8**	**> 4**	≤ 0.5	0.5	0.5	**> 128**	16	**> 128**	4	0.5	1
**AR89.2b**	Dog 89	Discharge	**> 64**	≤ 8	**> 8**	≤ 1	**2**	2^c^	**> 4**	≤ 0.5	0.5	0.5	**> 128**	16	2^c^	4	0.5	0.5
**AR94.2b**	Dog 94	Discharge	**> 64**	≤ 8	**> 8**	≤ 1	**> 2**	**8**	**> 4**	≤ 0.5	1	0.5	**> 128**	≤ 8	**> 128**	4	≤ 0.25	0.5
**AR96.2a**	Cat 96	Discharge	**> 64**	≤ 8	**> 8**	≤ 1	**2**	**8**	**> 4**	1	0.5	0.5	**> 128**	16	**> 128**	8	0.5	0.5
**AR135.2a**	Dog 135	Discharge	**> 64**	≤ 8	**> 8**	≤ 1	**> 2**	**16**	**> 4**	≤ 0.5	0.5	1	**> 128**	16	**> 128**	8	0.5	0.5
**AR144.2**	Dog 144	Discharge	**> 64**	≤ 8	**> 8**	≤ 1	**2**	2^c^	**> 4**	≤ 0.5	0.5	0.25	**> 128**	16	1	> 64	≤ 0.25	1
**AR145.2**	Cat 145	Discharge	**> 64**	≤ 8	**> 8**	≤ 1	**2**	**8**	**> 4**	≤ 0.5	0.5	0.25	**> 128**	16	**> 128**	4	≤ 0.25	1
**AR148.2**	Dog 148	Discharge	**> 64**	≤ 8	**> 8**	≤ 1	**2**	**8**	**> 4**	≤ 0.5	0.5	0.5	**> 128**	16	**> 128**	4	≤ 0.25	1
**AR76.3**	Cat 76	Follow-up	**> 64**	≤ 8	**> 8**	≤ 1	**2**	**8**	**> 4**	≤ 0.5	0.25	0.25	**> 128**	16	**> 128**	4	≤ 0.25	1
**AR96.3a**	Cat 96	Follow-up	**> 64**	≤ 8	**> 8**	≤ 1	**2**	**8**	**> 4**	≤ 0.5	0.5	0.25	**> 128**	16	**> 128**	4	≤ 0.25	1
**Number of non-susceptible strains**			**24**	**0**	**24**	**0**	**24**	**24**	**24**	**0**	**0**	**0**	**24**	**0**	**23**	**1**	**0**	**0**

AMP: ampicillin; CHL: chloramphenicol; CIP: ciprofloxacin; COL: colistin; ETP: ertapenem; FEP: cefepime; FOT: cefotaxime; GEN: gentamicin; IMI: imipenem; MERO: meropenem; MIC: minimum inhibitory concentration; NAL: nalidixic acid; SMX: sulfamethoxazole; TAZ: ceftazidime; TET: tetracycline; TGC: tigecycline; TMP: trimethoprim.

^a^ MICs highlighted in bold indicate resistance.

^b^ The resistance breakpoints presented here are those for *E. coli* from the European Committee on Antimicrobial Susceptibility Testing (EUCAST) [13], except for nalidixic acid, sulfamethoxazole and tetracycline, for which breakpoints from the Clinical and Laboratory Standards Institute (CLSI) [14] were used.

^c^ The MIC values of 2 μg/mL for cefepime and for ceftazidime were above the susceptible EUCAST breakpoint (S ≤ 1 μg/mL) and within the intermediate range (non-susceptible).

All isolates carried the carbapenemase gene *bla*_OXA-181_, which has been associated with a low level of resistance to carbapenems [4,17]. The isolates had MICs below the resistance breakpoints set by EUCAST for imipenem and meropenem but were resistant to ertapenem, with MICs above the resistance breakpoint (Table 2). All isolates also contained the *qnrS1* gene encoding a DNA gyrase protection protein which confers low-level resistance to fluoroquinolones, as well as a serine to leucine substitution at position 83 and an aspartic acid to asparagine substitution at position 87 in GyrA, and a serine to isoleucine substitution at position 80 in ParC. These amino acid substitutions are known to confer high-level resistance to fluoroquinolones in *E. coli* [1].

The pAmpC cephalosporinase *bla*_CMY-42_ was also found in all isolates, except in the two isolates (AR89.2b and AR144.2) which were not resistant to cefepime and ceftazidime. The tetracycline resistance found only in AR144.2 was associated with the tetracycline efflux gene *tet*(A). This isolate was also the only one that contained the *bla*_TEM-1B_ β-lactamase gene (Table 3).

**Table 3:** Characteristics of the carbapenemase-producing *Escherichia coli* isolates, Switzerland, May–August 2018 (n = 24)

Strains	ST^a^	Resistance phenotype^b^	Resistance genes^c^ and mutations^d^	Localisation^e^
AR8.2b; AR10.2b; AR17.2b; AR22.1; AR22.2; AR24.2b; AR25.2; AR26.2c; AR31.2a; AR33.2a; AR44.2; AR46.2b; AR62.2; AR76.2; AR76.3; AR81.2; AR94.2b; AR96.2a; AR96.3; AR135.2a; AR145.2; AR148.2 (n = 22)	410	AMP-CIP-ETP-FEP-FOT-NAL-TAZ	*bla*_CMY-42_, *sugE*	Plasmid IncI1, 47-kb
			*bla*_OXA-181_, *qnrS1*	Plasmid IncX3, 51-kb
			GyrA (Ser83Leu, Asp87Asn); ParC (Ser80Ile)	Chromosome
AR89.2b (n = 1)	410	AMP-CIP-ETP-FOT-NAL	*bla*_OXA-181_, *qnrS1*	Plasmid IncX3, 51-kb
			None	Plasmid IncI1, 88-kb^f^
			GyrA (Ser83Leu, Asp87Asn); ParC (Ser80Ile)	Chromosome
AR144.2 (n = 1)	410	AMP-CIP-ETP-FOT-NAL-TET	*bla*_OXA-181_, *qnrS1*	Plasmid IncX3, 51-kb
			*bla*_TEM-1B_, *tet*(A)	Plasmid IncI1, 98-kb^f^
			GyrA (Ser83Leu, Asp87Asn); ParC (Ser80Ile)	Chromosome

AMP: ampicillin; CHL: chloramphenicol; CIP: ciprofloxacin; COL: colistin; ETP: ertapenem; FEP: cefepime; FOT: cefotaxime; GEN: gentamicin; IMI: imipenem; MERO: meropenem; NAL: nalidixic acid; SMX: sulfamethoxazole; ST: sequence type; TAZ: ceftazidime; TET: tetracycline; TGC: tigecycline; TMP: trimethoprim.

^a^ Sequence type based on multilocus sequence typing was determined with MLSTtool 2.0.

^b^ Results interpreted according to the EUCAST and CLSI criteria (see Table 1).

^c^ Resistance genes were detected with ResFinder 3.0: *bla*_CMY-42_, cephalosporinase CMY-42 gene (resistance to FEP, TAZ); *bla*_OXA-181_, carbapenemase OXA-181 gene (resistance to AMP, ETP, FOT); *qnrS1*, DNA gyrase protection gene to fluoroquinolones (low-level resistance to fluoroquinolones); *bla*_TEM-1b_, β-lactamase TEM-1b gene (resistance to AMP); *tet*(A), tetracycline efflux gene (resistance to TET); *sugE*, quaternary ammonium compound resistance gene.

^d^ Chromosomal mutations were detected with ResFinder 3.0. GyrA (Ser83Leu and Asp87Asn), combined with ParC (Ser80Ile), mutations in the topoisomerase II and IV (high-level resistance to fluoroquinolones).

^e^ Incompatibility groups (Inc) were determined with PlasmidFinder 1.3.

^f^ As determined using plasmid SPAdes (v3.12.0).

### Characterisation of the plasmids containing resistance genes

**Figure 3:**
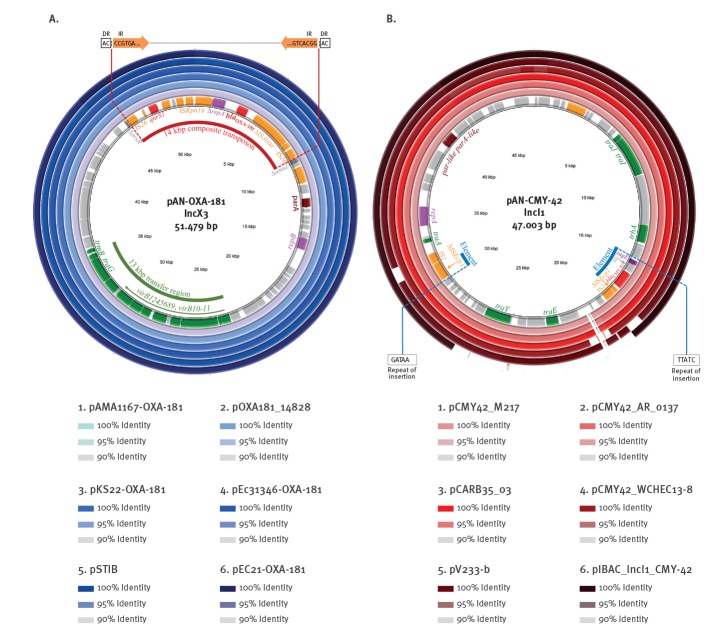
Circular maps of the resistance plasmids pAN-OXA-181 and pAN-CMY-42, Switzerland, May–August 2018 (n = 2)

The *bla*_OXA-181_ and *qnrS1* genes were co-localised on a 51 kb IncX3 plasmid (pAN-OXA-181) in strain AR24.2b (Figure 3). Plasmid mapping showed that all other 23 isolates also harboured this same plasmid. Plasmid pAN-OXA-181 was > 99.9% similar to other IncX3 plasmids from human *E. coli* strains in China (pOXA181_14828 and pEC21-OXA-181 [18,19], Denmark (pAMA1167-OXA-181) [20], and Lebanon (pSTIB) [21], as well as from a porcine *E. coli* strain in Germany (pEc31346-OXA-181) [22]. Plasmid pAN-OXA-181 differed by 3 SNPs from pKS22-OXA-181 which was detected in a *Klebsiella variicola* isolated from fresh vegetables imported into Switzerland [23] (Figure 3).

The coloured outer rings indicate regions of homology of the pAN-OXA-181 (GenBank accession number MK416154) and pAN-CMY-42 (GenBank acc. no. MK416155) plasmid of strain AR24.2b with other plasmids, based on BLASTn.

Left panel: pAN-OXA-181. The rings from the inner to the outer represent plasmids pAMA1167-OXA-181 (GenBank acc. no. CP024806; 99.99% identity, 1 single nucleotide polymorphism (SNP)), pOXA181_14828 (GenBank acc. no. KP400525; 100% identity), pKS22-OXA-181 (GenBank acc. no. KT005457; 99.98%, 3 SNP), pEc31346-OXA-181 (GenBank acc. no. KX894452; 99.99% identity, 1 SNP), pSTIB (GenBank acc. no. MG570092; 99.99% identity, 1 SNP) and pEC21-OXA-181 (GenBank acc. no. MG893567; 99.99% identity, 1 SNP).

Left panel: pAN-CMY-42. The rings from the inner to the outer represent plasmids pCMY42_M217 (GenBank acc. no. AP019190; 99% identity, 65% coverage), pCMY42_AR_0137 (GenBank acc. no. CP021882; 100% identity, 68% coverage), pCARB35_03 (GenBank acc. no. CP031656; 100% identity, 79% coverage), pCMY42_WCHEC13–8 (GenBank acc. no. KP789019; 100% identity, 71% coverage), pV233-b (GenBank acc. no. LC056425; 99% identity, 46% coverage) and pIBAC_IncI1_CMY-42 (GenBank acc. no. KY463221; 100% identity, 78% coverage).

The scale circle shows the coordinates in kbp of each plasmid. Genes are portrayed as coloured blocks in the inner ring of each circular map. Genes are coloured according to their classification. Red: antimicrobial resistance genes; dark purple: surface disinfectants resistance genes; orange: transposases; fuchsia: genes implicated in replication; green: transfer-associated genes; brown: partition genes; grey: other genes. The *Δumud* and *ΔarsR* genes are coloured in darker grey, flanking the composite transposon of pAN-OXA-181. The *blc* gene of pAN-CMY-42 is indicated in the map to better represent the composition of the native 4 kb element.

Both *bla*_OXA-181_ and *qnrS1* were found within a composite transposon in pAN-OXA-181, which was integrated between *umuD* and *asrR*, similarly to pOXA-181_14828 [18]. The composite transposon was flanked by two copies of the insertion sequence IS*26* which were oriented in the same direction. Two 50 bp inverted repeats IRL and IRR, each preceded by a 2 bp AC direct repeat, were found at both ends of the IS*26*-flanked composite transposon, which is indicative of a transposition event [19]. Plasmid pAN-OXA-181 also contained the replication gene *repB* and a 23 kb transfer region containing the *virB1–2–4–5–6–8–9–10–11*, *traG* and *trmB* genes (Figure 3).

Strain AR24.2b also contained a 47 kb IncI1 plasmid (pAN-CMY-42) which harboured the pAmpC gene *bla*_CMY-42_ (Figure 3). The *bla*_CMY-42_ gene is commonly found in plasmids within a 4 kb element composed by IS*Ecp1*, an outer membrane lipoprotein gene *blc* and a quaternary ammonium compound (QAC) resistance gene *sugE* (IS*Ecp1 – bla*_CMY-42_ *– blc – sugE*) [24]. In pAN-CMY-42 however, ΔIS*Ecp1* of this element was split by the insertion of a copy of IS*1*, and subsequent homologous recombination with another copy of IS*1* led to its modified structure, as previously described [24]. This recombination is supported by the inversion of the insertion repeats (GATAA and TTATC) (Figure 3). Plasmid pAN-CMY-42 also contains the replication gene *repA* and transfer genes *traA, traE, traJ, traI, traY* and *trbA*.

Plasmid pAN-CMY-42 differed from other CMY-42-containing IncI1 plasmids by its size and structure. The most closely related IncI1 CMY-42 plasmids deposited so far in GenBank were larger than pAN-CMY-42 by 1–50 kb, including plasmids isolated from canine *E.coli* in the UK (pCARB35_03) [16], human patients in Myanmar (pCMY42_M217) [25], China (pCMY42_WCHEC13–8) [24] and Italy (pIBAC_IncI1_CMY-42) [26], as well as from *E. coli* isolated from the environment (pV233-b) [27], and from an unknown source (pCMY42_AR_0137 (Figure 3).

Plasmid mapping of the other 23 *E. coli* showed that 21 isolates also contained this same plasmid (pAN-CMY-42), whereas the two isolates AR89.2 and AR144.2 lacked the 4 kb element (IS*Ecp1 – bla*_CMY-42_ *– blc – sugE*) on their IncI1 plasmid.

Plasmids pAN-OXA-181, pAN-CMY-42 and the chromosome of *E. coli* strain AR24.2b have been deposited in GenBank under accession numbers MK416154, MK416155 and CP035944, respectively.

## Discussion

**Figure 4:**
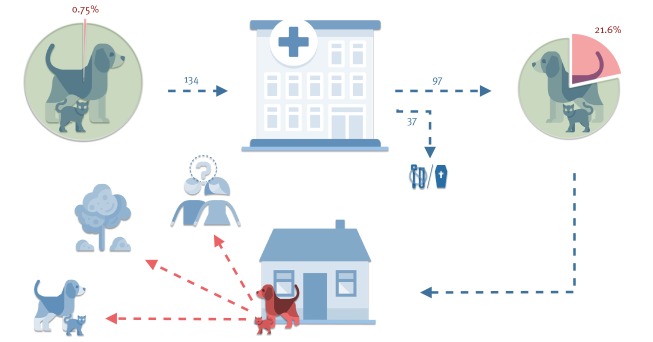
Screening of companion animals before and after hospitalisation, Switzerland, May–August 2018 (n = 134)

Surveillance of enteral carriage of antimicrobial-resistant bacteria in dogs and cats before and after hospitalisation in a companion animal hospital in Switzerland revealed the potential role of small animal clinics as an underestimated hotspot for acquisition of CP *E. coli*. In the particular situation presented here, almost one quarter of the hospitalised animals acquired a specific carbapenem-, cephalosporin- and fluoroquinolone-resistant clone of ST410 within a time span of 1-9 days of hospitalisation. The only dog found to be carrier of this clone at admission was chronologically not the first to be found positive during the study period, indicating that it was not the primary source of this strain. It may, however, have contributed to further enhance dissemination of the clone into the hospital. Long-term carriage beyond 108 days was only documented in one cat among the 10 dogs and two cats that were followed up. Nevertheless, all 21 animals that returned home with CP *E. coli* posed a potential risk of disseminating hospital-acquired CP *E. coli* into the environment and possibly to other animals and humans (Figure 4). Although the risk of CP Enterobacteriaceae transmission may be related to their relative abundance in faeces, our study was not set up to quantify the effective number of CP Enterobacteriaceae in faeces because it used enrichment and selective plates.

This figure shows the results of the screening of 134 animals at admission to the veterinary hospital and of 97 animals at discharge. It illustrates the potential risk for the environment, humans and other companion animals with regard to the carriage of CP *E. coli* by animals that have returned to their homes after having acquired CP *E. coli* during hospitalisation.

It is important to extend screening and detection methods in veterinary settings and diagnostic laboratories to detect such bacteria of medical and public health importance [4]. Detection of carbapenemases of the OXA-48 family may be challenging because of their low hydrolysing activity which specifies low MIC to carbapenems [4,5]. Application of low MIC screening values would allow identifying the OXA-181-producing *E. coli* isolates in our study since they exhibited MICs higher than the resistance breakpoint of > 0.5 μg/mL for ertapenem and higher than the meropenem screening cut-off value of > 0.125 μg/mL recommended by EUCAST [13].

Phylogenetic analyses of the CP *E. coli* using 3,778 genes of the core genome (cgMLST) confirmed high genetic relationship shared among all the *E. coli* isolates of ST410 from this study. This sequence type has recently been reported as a new emerging international high-risk clone, non-susceptible to critically important antibiotics such as fluoroquinolones, third-generation cephalosporins and carbapenems and with the potential of cross-sectorial transmission between wildlife, humans, pets and the environment [28,29]. *Escherichia coli* ST410 harbouring *bla*_OXA-181_ was first described in human infections in China in 2015 [18], then in Denmark in 2017 [20] and in Italy in 2018 [30], but so far it has not been reported in humans in Switzerland. These previously described human *E. coli* ST410 producing OXA-181 clustered into other branches of the cgMLST phylogenetic tree than the strain isolated in our study, indicating an independent origin. Another CP *E. coli* ST410 was identified in a dog in the UK in 2018 [16], GenBank accession number CP031653), emphasising the potential of ST410 for dissemination in the veterinary setting. This canine *E. coli* ST410 from the UK exhibited a slightly different cgMLST clustering and contained NDM-5 as carbapenemase rather than OXA-181. Although the two canine *E. coli* ST410 from the UK and Switzerland contained CMY-42 IncI1 plasmids which were highly similar, the one from Switzerland was lacking a 12 kb region compared with the CMY-42 IncI1 plasmid of the canine *E. coli* from the UK.

The present study revealed for the first time an OXA-181-producing *E. coli* ST410 associated with hospitalisation of companion animals. Carriage of carbapenemase-producing bacteria has so far not been reported among animals in Switzerland, but one study already reported OXA-181 in *K. variicola* isolated from imported fresh vegetables [23]. The presence of the same plasmid containing *bla*_OXA-181_ (pAN-OXA-181) in Enterobacteriaceae from companion animals, humans and vegetables suggest that interspecies transmission of this plasmid between *E. coli* and other Enterobacteriaceae is very likely. Nevertheless, the origin of the *E. coli* ST410 containing the carbapenemase plasmid pAN-OXA-181 in the veterinary setting is intriguing. Carbapenems are not used in this clinic and are therefore not likely to be the driving force for the selection and maintenance of this carbapenem-resistant *E. coli* ST410. The use of fluoroquinolones and β-lactams (Table 1) may have contributed to the selection of this ST410 clone or its plasmids, since the isolates also exhibited resistance to these classes of antimicrobials. That more animals were carriers of the CP ST410 clone at discharge than at admission as well as the high genetic relatedness of all isolates (including the identical plasmids) strongly indicate a common source of contamination within the hospital. Nevertheless, no cases of infection caused by an OXA-181-producing *E. coli* were revealed in dogs and cats during the 7 months following the outbreak using the same selective agar plates as those used in this study at the diagnostic unit of our institute. Of note, this referral hospital faced several cases of infections caused by a nosocomial clone of third-generation-cephalosporin-resistant *K. pneumoniae* ST11 producing the pAmpC DHA in 2010–2013 [31].

As an infection control measure, thorough disinfection of the hospital environment was performed after this outbreak in 2018 using disinfectant without QAC since ST410 also contained a QAC resistance gene (*sugE*) on the cephalosporinase plasmid pAN-CMY-42. Furthermore, stringent hospital hygiene and environmental cleaning protocols were introduced and a staff screening campaign was initiated. Staff training was also intensified to improve hand and environmental hygiene. The results and the impact of these measures are still being analysed and will be part of a follow-up study. Although no conclusion can be drawn at this stage of the study on the impact of possible colonisation of the staff or the pet owners, the results indicate that companion animals probably disseminated CP Enterobacteriaceae via faeces in the environment after hospitalisation and may represent a potential risk for transmission of CP Enterobacteriaceae to other animals and humans in the community and a serious One Health concern.

This outbreak stresses the need for national routine monitoring of carbapenemase-producing bacteria in companion animals using a representative sampling strategy analogous to the national surveillance of antibiotic resistance in food-producing animals at slaughterhouses in Switzerland and the European Union [32]. Based on the results of our study, we strongly recommend monitoring hospitalised companion animals for CP Enterobacteriaceae carriage at discharge using a sampling strategy representative of the number of hospitalised animals in the enrolled clinics. Such a surveillance programme should identify high-risk companion animal clinics and improve infection prevention and control as well as hygiene standards.

## Conclusion

This study provided evidences that dogs and cats can acquire CP Enterobacteriaceae within a short time of hospitalisation. Even if the colonisation of the animals did not last longer than 40 days in the majority of the animals, shedding of CP Enterobacteriaceae after hospitalisation represents a public health and environmental concern. Early detection and nationwide surveillance of carbapenemase-producing isolates, as well as effective infection prevention and control measures, need to be implemented in veterinary settings to limit the spread of high-risk clones in animals, humans and the environment.
